# Construction and immunogenicity of an mRNA vaccine against chikungunya virus

**DOI:** 10.3389/fimmu.2023.1129118

**Published:** 2023-03-15

**Authors:** Jingjing Liu, Xishan Lu, Xingxing Li, Weijin Huang, Enyue Fang, Wenjuan Li, Xiaohui Liu, Minglei Liu, Jia Li, Ming Li, Zelun Zhang, Haifeng Song, Bo Ying, Yuhua Li

**Affiliations:** ^1^ State Key Laboratory of Biotherapy and Cancer Center, West China Hospital, Sichuan University, Chengdu, China; ^2^ Division of Arboviral Vaccines, National Institutes for Food and Drug Control, Beijing, China; ^3^ Department of Preclinical Vaccine Research, Suzhou Abogen Biosciences Co., Ltd., Suzhou, China; ^4^ Division of HIV/AIDS and Sex-Transmitted Virus Vaccines, National Institutes for Food and Drug Control, Beijing, China

**Keywords:** chikungunya virus, mRNA vaccine, immunogenicity, sequential immunization, prime-boost

## Abstract

Chikungunya fever (CHIKF) has spread to more than 100 countries worldwide, with frequent outbreaks in Europe and the Americas in recent years. Despite the relatively low lethality of infection, patients can suffer from long-term sequelae. Until now, no available vaccines have been approved for use; however, increasing attention is being paid to the development of vaccines against chikungunya virus (CHIKV), and the World Health Organization has included vaccine development in the initial blueprint deliverables. Here, we developed an mRNA vaccine using the nucleotide sequence encoding structural proteins of CHIKV. And immunogenicity was evaluated by neutralization assay, Enzyme-linked immunospot assay and Intracellular cytokine staining. The results showed that the encoded proteins elicited high levels of neutralizing antibody titers and T cell-mediated cellular immune responses in mice. Moreover, compared with the wild-type vaccine, the codon-optimized vaccine elicited robust CD8^+^ T-cell responses and mild neutralizing antibody titers. In addition, higher levels of neutralizing antibody titers and T-cell immune responses were obtained using a homologous booster mRNA vaccine regimen of three different homologous or heterologous booster immunization strategies. Thus, this study provides assessment data to develop vaccine candidates and explore the effectiveness of the prime-boost approach.

## 1 Introduction

The chikungunya virus (CHIKV) is a pathogen that causes chikungunya fever (CHIKF) in humans; *Aedes aegypti* and *A. albopictus* are its hosts. Symptoms, such as high fever, rash, and headache, appear in the acute phase after infection, and there is a high incidence of joint pain in the chronic phase (1). In recent decades, CHIKF has spread to over 100 countries worldwide, thereby leading to a number of epidemics (2). Some infected patients suffer from arthralgia for years or even decades after infection, even though other symptoms have disappeared (3). This causes great suffering to patients and serious social and economic development problems. CHIKV has four genotypes, but is generally believed to have only one serotype; therefore, a vaccine developed using one genotype should achieve cross-protection against all genotypes (4). Although this simplifies the development of a CHIKV vaccine to some extent, no vaccine has yet been approved. As Arboviral diseases continue to receive international attention, several CHIKV vaccines have been developed and are in clinical trials, including inactivated vaccines (5, 6), live-attenuated vaccines (LAV) (7), recombinant vector vaccines (8, 9), virus-like particle (VLP) vaccines (10), and mRNA vaccines (11).

In just three decades since their large-scale deployment, mRNA vaccines have become one of the most important vaccine strategies to address infectious disease epidemics owing to their simplicity, short process cycle, and less restrictive manufacturing environment. Several mRNA vaccines have been approved for the market or are in clinical trials (12). For instance, mRNA-1273 from Moderna and BNT162b2 from BioNTech were the first coronavirus disease 2019 (COVID-19) vaccines approved for use in several countries worldwide and have been shown to have broad immunogenicity (13–16). In addition, clinical trials for various prime-boost strategies have been initiated. Homologous prime-boost immunization strategies are used with most vaccines to achieve good immunity. Some vaccines, including vaccines against human immunodeficiency viruses (HIVs), Ebola virus disease (EVD), malaria, tuberculosis, influenza, and hepatitis B, have undergone heterologous prime-boost investigation with the expectation of improving vaccine-induced immunity. A mix-and-match immunity approach combined an Ebola adenovirus vector and a modified vaccinia virus Ankara (MVA) vector to produce a vaccine, which has exhibited good safety and immunogenicity in clinical trials (17). However, this technique has not been successful for HIV or malaria vaccines (18, 19). A heterologous prime-boost clinical program for COVID-19 is underway and includes different combinations of the Moderna, Pfizer, AstraZeneca, Johnson & Johnson, Sinopharm, and Sinovac vaccines (20).

In this study, we constructed a CHIKV vaccine using an mRNA vaccine platform. We used a structural protein gene of CHIKV as the target antigen gene, which is similar to recombinant vectored vaccine and mRNA vaccine candidates under preclinical development (2). The expression of the entire structural cassette polyprotein promotes folding of the correct antigen and resembles the proper formation that mimic the protein structure of CHIKV (21). mRNA is obtained by *in vitro* transcription and self-assembly formulation with lipid nanoparticles (LNPs), which are then purified to prepare the vaccine. The immunogenicity of the vaccine was evaluated in mice. In a previous study, we constructed a recombinant CHIKV vaccine that expressed the same antigen as the mRNA vaccine using a replication-defective human type 5 adenovirus (Ad5) as the vector, which also exhibited a good immune response. We used the recombinant adenovirus vector vaccine and mRNA vaccine in a prime-boost strategy of sequential immunization, with the aim of further improving efficacy.

## 2 Materials and methods

### 2.1 Cells and virus

Both 293T and DC 2.4 cells were maintained in DMEM (Gibco) supplemented with 10% heat-inactivated fetal bovine serum (FBS, Gibco) and penicillin–streptomycin (100 U/ml, Gibco). Pseudovirus pSG-cmv-flu-chikv (22) was stored at −80°C and titrated using the 50% tissue culture infectious dose (TCID_50_) assay in 293T cells.

### 2.2 mRNA preparation and formulation

The cDNAs encoding wild-type (WT) or codon-optimized (OP) structural protein C-E3-E2-6K-E1 genes from CHIKV strain LR2006 OPY1 (23) were cloned into plasmid ABOP-028 (GENEWIZ), which contained 5′ and 3′ untranslated regions (UTRs) and a poly A tail. The mRNA was synthesized *in vitro* using T7 polymerase-mediated transcription from the linearized plasmid DNA template.

Formulation was performed as described previously for the COVID-19 vaccine (24). Briefly, lipids were dissolved in ethanol containing an ionizable lipid, 1,2-distearoyl-sn-glycero-3-phosphocholine (DSPC), cholesterol, and PEG-lipid (molar ratios of 50:10:38.5:1.5). The lipid mixture was run through a T-mixer with mRNA dissolved in 20 mM citrate buffer (pH = 4.0) at a ratio of 1:2. The generated mRNA-LNPs were diafiltrated against PBS (pH = 7.4) in a dialysis cassette with 20 kD MWCO overnight, passed through a 0.22-μm filter, and stored at 2°C–8°C until use. The product was characterized for particle size and distribution using a particle size analyzer (Malvern Panalytical), RNA concentration using HPLC (Thermo Fisher Scientific), and encapsulation using RiboGreen reagent (Thermo Fisher Scientific).

### 2.3 mRNA transfection

DC 2.4 cells were seeded in 24-well plates at 500,000 cells/well and then transfected with 1 μg of mRNA to form cell monolayers using Lipofectamine 2000 Transfection Reagent (Thermo Fisher Scientific). After 6 h, the medium was replaced with DMEM supplemented with 3% FBS. Following 24 h post-transfection, cells were fixed with ice-cold acetone for 10 min and incubated with a polyclonal mouse anti-CHIKV antibody (prepared in our laboratory) and then FITC-labeled goat anti-mouse IgG (Abcam). Nuclei were counterstained with DAPI (Thermo Fisher Scientific). Fluorescence was observed using a fluorescent microscope (Olympus).

After 16–26 h post-transfection, cells were lysed with RIPA buffer plus proteinase inhibitor (Sigma), clarified by centrifugation at 12,000 × *g*, and then 5 × SDS loading buffer was added, and cells were kept at 95°C for 10 min. The lysates were run on a 10% NuPAGE Bis-Tris gels (Invitrogen) followed by transferring proteins to a nitrocellulose membrane. The membrane was incubated with a polyclonal mouse anti-CHIKV antibody (prepared in our laboratory) and HRP-labeled goat anti-mouse IgG (Abcam). DC 2.4 cells with lysing buffer and E2 protein were used as negative and positive controls (ProSpec), respectively. Blots were developed using ECL reagents (GE).

### 2.4 Animal experiments

Female C57BL/6 mice were obtained from the Institute for Laboratory Animal Resources at the National Institutes for Food and Drug Control (NIFDC). For neutralizing antibody responses, mice aged 6–8 weeks were injected *via* an intramuscular (im) route with 100 μl of the indicated vaccine or negative control. Serum was collected from the submandibular vein before and/or post-prime immunization. For cellular immune responses, mice aged 6–8 weeks were immunized *via* the im route with 100 μl of the indicated vaccine or negative control. Spleen tissues were collected after immunization. Housing and experimentation of mice were performed strictly in accordance with the guidelines set by the Association for the Assessment and Accreditation of Laboratory Animal Care (AAALAC). The study protocol was approved by the Animal Care and Use Committee of the NIFDC.

### 2.5 Homologous and heterogeneous prime-boost

The vaccines used in this experiment were the mRNA vaccine and Ad5 vaccine (developed in our laboratory). Both vaccines contain the prototype gene of structural protein. Mice were immunized using different vaccination regimens: same vaccination routes (2×Ad5, 2×mRNA), different vaccination routes (Ad5 + mRNA), and negative control groups. The prime vaccination day was set as day 0, and the boost was performed on day 14. Serum and spleen tissues were collected before and after prime immunization.

### 2.6 Neutralization assays

The neutralization assay using pseudovirus was similar to that used for severe acute respiratory syndrome coronavirus 2 (SARS-CoV-2) as described previously (25). Serial threefold diluted heat-inactivated serum obtained from C57BL/6 mice was incubated with 400 TCID_50_ of the pseudovirus for 1 h at 37°C. The 293T cells were added to serum–virus complexes in 96-well plates at 50,000 cells/well and incubated for approximately 48 h, together with the virus control and cell control in wells. The supernatant was then removed and luciferase substrate (Perkin Elmer) was added to each well, followed by incubation for 2 min. Luminescence was measured for pseudovirus titration. The 50% inhibitory dilution (EC_50_) was defined as the serum dilution at which the relative light units (RLUs) were reduced by 50% compared with the virus control after subtraction of the background RLUs in control cells.

### 2.7 Enzyme-linked immunospot assay

Spleens were isolated from mice and dispersed in a 40-μm cell strainer with mouse lymphocyte separation medium (Dakewe). The tissues were centrifuged at 800 × *g* for 20 min and covered with 1 ml of RPMI-1640 medium (Hyclone). After centrifugation, splenocytes were resuspended in serum-free medium (Dakewe). Interferon gamma (IFN-γ)- or interleukin-2 (IL-2)-positive cells were assessed using precoated enzyme-linked immunospot (ELISpot) kits (MabTech) according to the manufacturer’s protocol. Briefly, 96-well plates were blocked with RPMI-1640 medium containing 10% FBS for at least 2 h at 24°C. The splenocytes were transferred to the wells at 500,000 cells/well and stimulated at 37°C for 18 h with Ad5 containing entire structural proteins C-E3-E2-6K-E1. Phorbol ester (PMA)/Ionomycin (Dakewe) and RPMI-1640 media were used as the positive and blank control, respectively. The plates were incubated with anti-mouse IFN-γ or IL-2 antibody for 2 h and streptavidin-horseradish peroxidase for 1 h at ~25°C. The visualized immunoprecipitate revealed that the following treatment with TMB substrate solution was subsequently imaged and quantified using an ImmunoSpot S6 Universal instrument (Cellular Technology Limited).

### 2.8 Intracellular cytokine staining

Splenocytes were transferred into wells at 500,000 cells/well and stimulated at 37°C for 8 h with Ad5 containing entire structural proteins C-E3-E2-6K-E1. Brefeldin A (BioLegend) was then added to block cytokine secretion and incubation continued for 4 h. Following two washes with PBS, splenocytes were incubated with the following antibodies against lineage markers: PE anti-mouse CD3e, FITC anti-mouse CD4, and PerCP/Cy 5.5 anti-mouse CD8a (all from BioLegend). After two washes with PBS, cells were fixed, permeabilized with Cytofix/Cytoperm (BD Biosciences), and incubated with the following antibodies against intracellular markers: APC anti-mouse IFN-γ and PE/Cy7 anti-mouse IL-2 (all from BioLegend). Cells were analyzed using a FACS Lyric analyzer (BD Biosciences).

### 2.9 Statistical analysis

Data were plotted and analyzed using GraphPad Prism 8.0 and presented as geometric means ± geometric standard deviation. Neutralization antibody levels were compared by two-way analysis of variance (ANOVA), and an unpaired *t*-test was used for comparison of cellular immune levels. A *p*-value of <0.05 was considered statistically significant (*p < 0.05; ** *p* < 0.01; *** *p* < 0.001; **** *p* < 0.0001; ns, no significance).

## 3 Results

### 3.1 Construction of mRNA vaccine encoding a CHIKV structural protein

**Figure 1:**
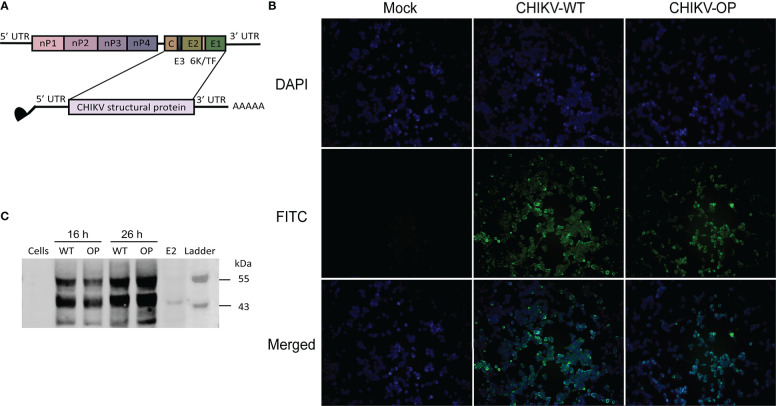
Design and protein expression of the CHIKV mRNA vaccine. **(A)** Schematic of the CHIKV genome structure and mRNA construct. **(B)** Structural protein expression following mRNA transfection with wild-type (WT) or codon-optimized (OP) mRNA as determined using FITC immunofluorescence staining (scale bar, 400 μm). **(C)** Western blot analysis of structural protein expression from CHIKV mRNA-WT or -OP in DC 2.4 cells and lysate at 16 or 26 h post-transfection. E2, control structural protein.

The structural proteins C-E3-E2-6K-E1 of WT CHIKV virus strain LR2006 OPY1 were selected as the target antigens for the mRNA coding sequence. Moreover, 5′ and 3′ UTRs were added to increase mRNA stability and regulation of translation. A poly(A) tail of ~100–130 bp was synthesized to follow the 3′ UTR. Transcription of the linearized DNA template to mRNA was mediated by the T7 promoter, and the complete mRNA was obtained by adding the Cap-1 structure (N7^m^GpppG^m^) to the 5′ end of the RNA using a vaccinia capping enzyme. We also constructed an mRNA-OP using OP sequences (26) (**Figure 1A**) and a sequence optimization strategy for mRNA vaccines as previously described (24). The resulting mRNA was transfected into DC 2.4 cells. Immunofluorescence assays using anti-CHIKV structural protein polyclonal antibodies indicated that the mRNA-WT and -OP translated targeted proteins were expressed intracellularly and on the cell membrane (**Figure 1B**). Cell lysates were collected, immunoblotting assays were performed using anti-CHIKV structural protein polyclonal antibodies, and specific protein expression was detected in both mRNA-WT and -OP transfected cell samples (**Figure 1C**). mRNA was encapsulated in LNPs for diafiltration and purification; formulation with an average particle size of ~79 nm and an encapsulation rate of over 90% were obtained (**Table S1**, **Figure S1**).

### 3.2 Neutralizing antibody analysis

**Figure 2:**
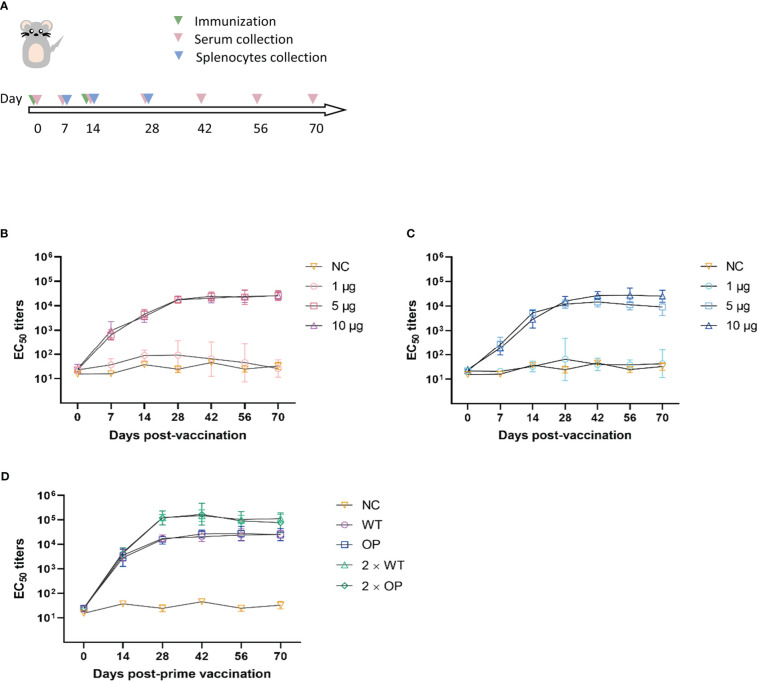
Neutralizing antibody responses to the CHIKV mRNA vaccine in mice. **(A)** Mice were immunized with one or two doses, and serum and splenocytes were collected at the indicated time points. EC_50_ values were assessed using CHIKV pseudovirus. **(B–D)** Post-vaccination neutralization curves of EC_50_ following **(B)** a single dose of wild-type (WT) vaccine, **(C)** a single dose of codon-optimized (OP) vaccine, and **(D)** prime-boost vaccines. *n* = 6 mice per group; one data point represents the geometric mean titer per group at each time point. Bars represent geometric means ± geometric standard deviation (SD). NC, negative control.

In the single-dose immunization regimen, C57BL/6 mice received an im injection of 1, 5, or 10 μg WT or OP vaccine, with PBS solution as the negative control. Serum was collected before immunization and on days 7, 14, 28, 42, 56, and 70 post-immunization for neutralizing antibody testing using a pseudovirus assay (**Figures 2A–C**). For the 1-μg WT dose, the EC_50_ antibody titer was maintained at a low level approaching negative control; the EC_50_ antibody titer exhibited a high agreement between that of the 5- and 10-μg doses (*p* = 0.9958), which reached ~20,000 on day 42 post-immunization and remained steady. At the 1-µg OP dose, antibody was not induced; at the 10 μg dose, the EC_50_ antibody titer reached a peak of 26,833 on day 42 post-immunization, which was approximately twice that of the 5-μg dose, although the EC_50_ antibody titer showed no significant difference with the 10-μg dose of OP (*p* = 0.0827). The neutralizing antibody titers of the WT vaccine were better than those of the OP vaccine at the 5-μg dose, which suggests that base changes in the target genes may have affected humoral immunity.

In the booster immunization regimen, WT and OP vaccines were given at a dose of 10 μg and boosted with an equal dose of vaccine on day 14 post-initial immunization. Serum was collected before immunization and on days 7, 14, 28, 42, 56, and 70 post-prime immunization for neutralizing antibody testing (**Figure 2D**). Neutralizing antibody levels for both vaccines increased substantially on day 28 post-immunization, with a greater than sixfold increase compared with that of single-dose immunization. Furthermore, EC_50_ antibody titers reached over 100,000, peaking on day 42 post-immunization for the WT and OP vaccines. Neutralizing antibody titers were significantly higher following booster immunization than single-dose immunization (WT: *p* < 0.0001; OP: *p* = 0.0028). The results indicate that booster immunization with both WT and OP vaccines induced relatively high levels of neutralizing antibodies in mice.

### 3.3 Cellular immunity response

C57BL/6 mice were immunized with a single dose of 10 μg of WT or OP vaccine, with PBS solution as the negative control. Splenocytes were harvested from the mice on days 7, 14, and 28 after immunization (**Figure 2A**) and stimulated with Ad5 containing CHIKV structural proteins; ELISpot and intracellular cytokine staining (ICS) assays were performed.

**Figure 3:**
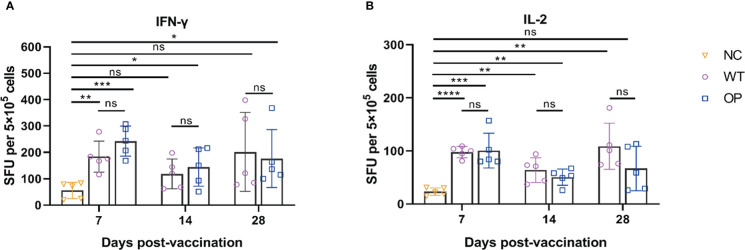
CHIKV-specific T-cell responses induced by CHIKV mRNA vaccine. mRNA vaccine was administered to mice and the splenocytes were harvested and analyzed. **(A)** IFN-γ-secreting cells and **(B)** IL-2-secreting cells were quantified by ELISpot assay. *n* = 5 mice per group; each data point represents the mean number of spots per well. * *p* < 0.05; ** *p* < 0.01; *** *p* < 0.001; **** *p* < 0.0001; ns: *p* > 0.05. NC, negative control; SFU, spot forming units; WT, wild-type vaccine; OP, codon-optimized vaccine.

The levels of IFN-γ and IL-2 secretion by splenocytes were analyzed by the ELISpot assay (**Figure 3**). The WT and OP vaccines induced strong IFN-γ (WT: *p* = 0.0025; OP: *p* = 0.0002) and IL-2 (WT: *p* < 0.0001; OP: *p* = 0.0009) levels on day 7 post-immunization compared with the negative control group. There was no statistically significant difference in the levels of cytokine-secreting cell number between the WT and OP vaccine groups (IFN-γ: *p* = 0.1484; IL-2: *p* = 0.8606). Furthermore, both vaccines displayed stable cellular responses within 28 days post-immunization.

**Figure 4:**
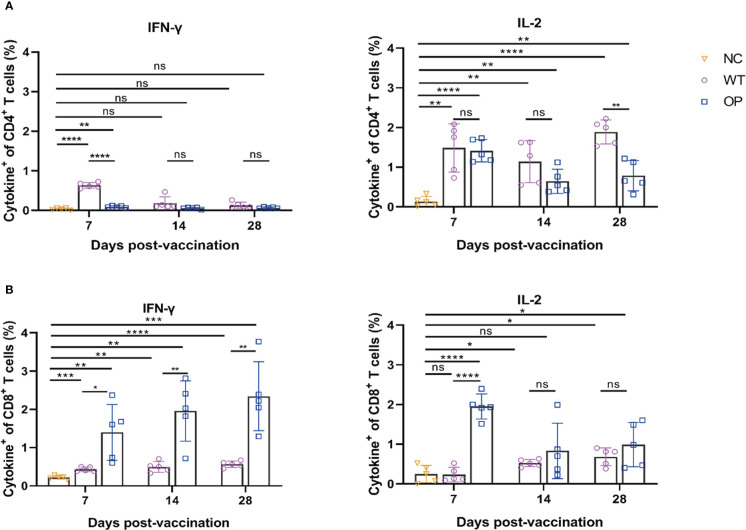
CD4^+^ and CD8^+^ T-cell responses induced by CHIKV mRNA vaccine. mRNA vaccine was administered to mice and the splenocytes were harvested and analyzed. Percentage of intracellular **(A)** IFN-γ and IL-2 CD4^+^ T cells, and **(B)** CD8^+^ T cells as determined by ICS. *n* = 5 mice per group; each data point represents the mean number of spots per well. * *p* < 0.05; ** *p* < 0.01; *** *p* < 0.001; **** *p* < 0.0001; ns: *p* > 0.05. NC, negative control; WT, wild-type vaccine; OP, codon-optimized vaccine.

Both mRNA vaccines induced CD4^+^ and CD8^+^ T-cell responses (**Figure 4**). However, the WT vaccine induced IFN-γ CD4^+^ T-cell response on day 7, but not on day 14 and day 28. Interestingly, IFN-γ CD4^+^ T cells were not clearly detected or were detected at very low levels in the OP vaccine group. In contrast, both vaccines induced IL-2 CD4^+^ T-cell responses with the OP vaccine group having a lower T-cell number on day 28 (*p* = 0.0010) than the WT vaccine. The OP vaccine induced a higher IFN-γ CD8^+^ T-cell response than the WT group. The latter showed a very low level of IFN-γ CD8^+^ T-cell responses. The IL-2 T-cell response showed a similar trend with the OP group having a higher IL-2 CD8^+^ T-cell response although the differences between the two groups were not significant on day 14 (*p* = 0.3622) and day 28 (*p* = 0.2874).

### 3.4 Homologous and heterologous prime-boost

To compare the immune responses of the different prime-boost regimens, we used the Ad5 CHIKV vaccine constructed in our previous study, which was obtained by cloning the complete CHIKV structural protein genes (C-E3-E2-6K-E1) into a shuttle plasmid. We employed homologous recombination with the plasmid using the AdEasy adenovirus packaging system, transfecting Ad293 cells and assembling them into a mature recombinant adenovirus, which was amplified and purified to obtain the Ad5 vaccine expressing the CHIKV antigen.

**Figure 5:**
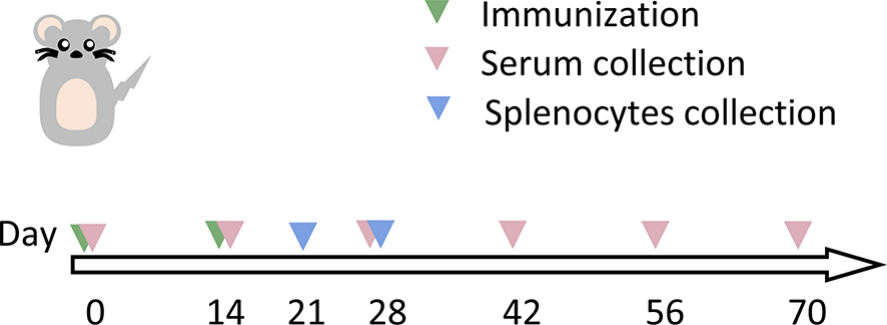
Schematic representation of the schedule for immunization and immunological characterization of the different immunization regimens. Mice were immunized with two doses of vaccine (as indicated in **Table 1**), and serum and splenocytes were collected at the indicated time points. NC, negative control; PBS, phosphate-buffered saline; Ad5, replication-defective human type 5 adenovirus-vectored vaccine.

As illustrated for the immunization regimen in **Figure 5**, mice were immunized using different vaccination regimens: prime-boosted homologously with mRNA vaccine, or with Ad5 vaccine, or primed with Ad5 vaccine followed by heterologous boosting with mRNA vaccine at a 14-day interval. The overall scheme for group design and immunization is shown in **Table 1**. A total of 10^7^ infectious units (IFU) of Ad5 vaccine or 10 μg of mRNA vaccine was used for vaccination.

**Table 1:** Schedule for different immunization regimens.

Group	Prime	Boost
NC	PBS	PBS
2×mRNA	mRNA	mRNA
2×Ad5	Ad5	Ad5
Ad5+mRNA	Ad5	mRNA

Details of the different prime-boost strategies used to immunize four groups of mice. NC, negative control; PBS, phosphate buffered solution; mRNA, mRNA vaccine; Ad5, replication-defective human type 5 adenovirus-vectored vaccine.

**Figure 6:**
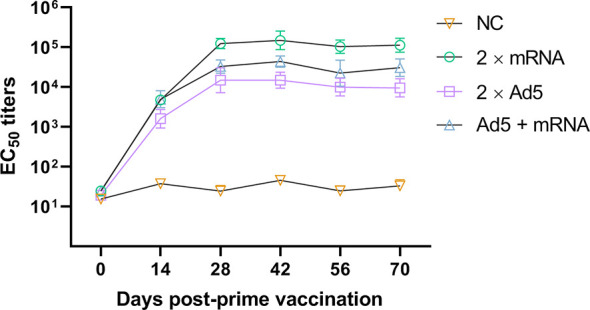
Neutralizing antibody responses to different immunization regimens. Mice in three groups were immunized with different vaccine combinations including homologous immunization with the developed mRNA vaccine, replication-defective human type 5 adenovirus-vectored vaccine (Ad5) vaccine, and priming immunization with Ad5 vaccine followed by mRNA vaccine. Neutralizing EC_50_ values were assessed using the CHIKV pseudovirus. *n* = 6 mice per group, one data point represents the geometric mean titer per group at each time point. Bars represent geometric means ± geometric standard deviation (SD). NC, negative control.

Serum was collected on day 0 before immunization and on days 14, 28, 42, 56, and 70 post-prime immunization (**Figure 6**). The EC_50_ antibody titers of all immunization groups peaked on day 42 post-prime immunization. The EC_50_ antibody titer of the 2×mRNA immunization group was 149,428, which was significantly higher than that of the Ad5 + mRNA group (*p* = 0.0004) and the 2×Ad5 group (*p* < 0.0001), and it was maintained above 100,000 thereafter. There was a statistically significant difference in the titers between the Ad5+mRNA and 2×Ad5 groups (*p* = 0.0014), with peak antibody titers of 43,679 and 13,335, respectively. No significant increase in neutralizing antibody titers was observed with heterologous immunization compared to the homologous regimen.

**Figure 7:**
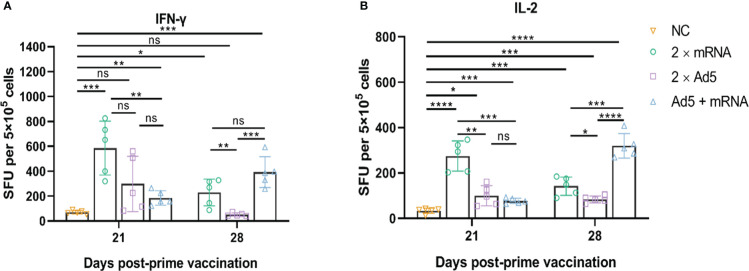
CHIKV-specific T-cell responses induced by different immunization regimens. Mice in three groups were immunized with different prime-boost strategies and splenocytes were harvested and analyzed. **(A)** IFN-γ-secreting cells and **(B)** IL-2-secreting cells were quantified by ELISpot assay. *n* = 5 per group; each data point represents the mean number of spots from wells. * *p* < 0.05; ** *p* < 0.01; *** *p* < 0.001; **** *p* < 0.0001; ns: *p* > 0.05. NC, negative control; SFU, spot forming units; Ad5, replication-defective human type 5 adenovirus-vectored vaccine.

Splenocytes were harvested on days 21 and 28 post-prime immunization, and ELISpot and ICS assays were performed. The ELISpot assay (**Figure 7**) showed that the 2×Ad5 group failed to induce IFN-γ (day 21: *p* = 0.0519; day 28: *p* = 0.0561), but inducing IL-2 response (day 21: *p* = 0.0117; day 28: *p* = 0.0003), while IFN-γ and IL-2 were induced in the other two groups (2×mRNA and Ad5+mRNA). On day 21 post-prime immunization, the cytokine levels in the 2×mRNA group were higher than those in the other two groups with a statistically significant difference compared with the Ad5 + mRNA group (IFN-γ: *p* = 0.0041; IL-2: *p* = 0.0002). On day 28 post-initial immunization, the cytokine levels in the Ad5+mRNA group were significantly higher, while they decreased in the other two groups. Moreover, cytokine levels in the Ad5+mRNA group showed a statistically significant difference from those in the 2×Ad5 group (IFN-γ: *p* = 0.0003; IL-2: *p* < 0.0001); IL-2 levels were higher than those in the 2×mRNA group (*p* = 0.0004), while the Ad5+mRNA group showed similar levels of IFN-γ induction to those of the 2×mRNA group (*p* = 0.0564).

**Figure 8:**
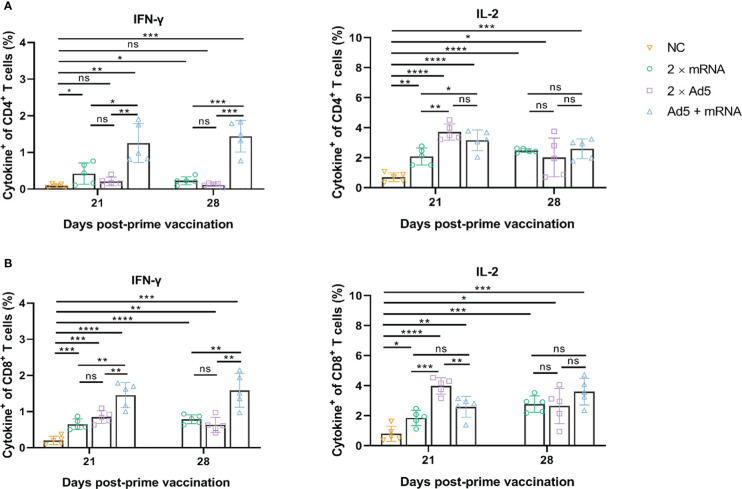
CD4^+^ and CD8^+^ T-cell responses induced by different immunization regimens. Mice in four groups were immunized with different prime-boost strategies and splenocytes were harvested and analyzed. Percentage of intracellular **(A)** IFN-γ and IL-2 positive CD4^+^ T cells, and **(B)** CD8^+^ T cells determined by ICS. *n* = 5 per group; each data point represents the mean number of spots per well. * *p* < 0.05; ** *p* < 0.01; *** *p* < 0.001; **** *p* < 0.0001; ns: *p* > 0.05. NC, negative control; Ad5, replication-defective human type 5 adenovirus-vectored vaccine.

ICS results (**Figure 8**) showed that CD4^+^ and CD8^+^ T cells in the Ad5+mRNA group exhibited higher levels of induced IFN-γ on both day 21 and day 28 post-prime immunization, while IFN-γ in the other two groups was induced primarily by CD8^+^ T cells. CD4^+^ and CD8^+^ T cells in all three immunization groups induced comparable levels of IL-2 on day 28 post-prime immunization.

## 4 Discussion

Here, we developed an mRNA vaccine against CHIKV encoding structural proteins (C-E3-E2-6K-E1) of the highly infectious LR2006 OPY1 strain from the IOL lineage (23). The vaccine was based on the COVID-19 mRNA-LNP vaccine platform (24), which has been shown to have satisfactory safety and efficacy in clinical trials (27).

The genes encoding CHIKV structural proteins can be successfully expressed in cells and the mRNA vaccine induced high-level titers of neutralizing antibodies and strong cellular immune responses in mice. Humoral immunity plays a crucial role in protection against CHIKV infection (28, 29). Neutralizing antibody titers reached up to 169,564 when mice were immunized with two doses of WT vaccine (10 μg) and remained at high levels. In a previous publication by Campos, two-dose immunization with the ChAdOx1 Chik vaccine candidate resulted in a neutralizing antibody titer of 15,300, and a single-dose immunization with the vaccine protected mice from a lethal viral attack (21, 30). The neutralizing antibody titers induced by the mRNA vaccine in the current study were much higher than those reported by Campos, indicating that the mRNA vaccine may have the potential to confer sufficient protection efficacy.

In respect to cellular immunity, single-dose immunization (WT, 10 μg) induced IFN-γ and IL-2 on day 7 and maintained high cytokine levels. IFN-γ was induced primarily by CD8^+^ T cells, and IL-2 was induced by CD4^+^ and CD8^+^ T cells. Although a strong T-cell immune response was induced, the role of cellular immunity in regulating CHIKV virus replication and clearance requires further investigation. Studies have shown that CD4^+^ and CD8^+^ T cells can induce a long-lasting immune response after CHIKV infection, reducing viral replication capacity, but they do not appear to protect against acute CHIKV infection (31). Moreover, virus clearance does not solely depend on CD4^+^ and CD8^+^ T cells in a mouse model (32).

The OP mRNA vaccine in the study induced less persistent neutralizing antibodies than the WT vaccine, while cellular immunity, especially IFN-γ secreted by CD8^+^ T cells, was significantly higher than that observed from the WT vaccine group. mRNA can directly interact with some pattern recognition receptor to stimulate the innate immune response. It was reported that mRNA-induced upregulation of type-I IFN tends to upregulate CD8^+^ T-cell cytotoxicity, attenuating the helper T cells’ effect in humoral immune responses. As a consequence, it may lead to reduced antigen expression and diminished vaccine efficacy (33, 34). Codon optimization is thought to influence mRNA translation and stability (35). However, rapid translation could prevent adequate folding of the target protein, as shown in a codon-optimized firefly luciferase mRNA that reduced the expression capacity of fluorescent proteins (36). In addition, highly stable secondary structures may hinder ribosome binding, and may be recognized and eliminated by the innate immune response (37). We speculated that the OP vaccine may enhance a stronger innate immune response, allowing activated CD8^+^ T cells to clear the mRNA, thereby limiting the translation of effective antigens and leading to a decrease in neutralizing antibodies. Only the larger dosage (10 μg) could achieve the same results as that of the WT vaccine. Thus, how to reach a balance between innate and adaptive immunity that allows mRNA to serve as an adjuvant, while also allowing effective translation, is a major goal in mRNA vaccine development.

We conducted a study on the immunogenicity of homologous and heterologous booster immunization to evaluate whether we could effectively improve the immune response to mRNA vaccines. Heterologous booster immunization did not result in higher neutralizing antibody titers; instead, homologous booster immunization with mRNA vaccine yielded better neutralizing antibody titers than the other groups. With respect to the cellular immune response, heterologous booster immunization showed delayed kinetics in the induction of high levels of cytokines, while CD4^+^ and CD8^+^ T cells induced more balanced levels of cytokines. Moreover, homologous booster immunization with the mRNA vaccine still exhibited a stronger T-cell immune response, and homologous immunization with the Ad5 vaccine induced more stable levels of cytokines over 28 days. This result differs from that of the COVID-19 vaccine in that the first dose of ChAdOx1-S, followed by an mRNA vaccine, elicited higher anti-COVID-19 neutralizing antibody titers and T-cell immune responses in mouse models (38). The sequence of administering ChAdOx1-S in the first dose followed by the mRNA vaccine in the second dose yielded higher immunogenicity than the reverse order of immunization (39, 40). The results of ELISpot and ICS appear to be inconsistent, as previously reported (41, 42). This may be related to the characteristics of the two methods. ELISpot is better able to measure low-level responses owing to its level of sensitivity. However, ICS allows for discrimination and phenotypic analysis of individual cytokine-producing cells across a broad dynamic range (41). A previous finding showed that peptides were more effective in stimulating responses than whole-protein antigens (43). To ensure a cellular response, a combination of several methods should be used. For most vaccines, booster immunization results in more effective protection. Activation of innate immunity leads to specific antibody production and memory T cells after primary immunization; re-activation of innate and adaptive immunity results in enhanced antigen presentation, cytokine secretion, and other functions that further promote differentiation of secondary memory cells after booster immunization (44). Several aspects may influence the immune response in heterologous booster immunity. For example, different properties of vectors may induce different immune responses, different immunization regimens may lead to attenuation of anti-vector immunity, and different antigens may produce different effects in induced T-cell immune responses (45). More data are needed to support the safety and efficacy of heterologous booster immunization.

Our study has several limitations. First, we did not measure the vaccine protection against a lethal challenge and viremia because of the limited resources because live CHIKV experiments should be performed in a biosafety level 3 laboratory or biosafety animal level 3 laboratory, and protection against CHIKV challenge needs to be confirmed in further studies. Neutralization of the antibody titer induced by the mRNA vaccine was superior to that of the ChAdOx1 Chik vaccine, which could protect mice from a lethal viral attack. Second, we used Ad5 containing entire structural proteins C-E3-E2-6K-E1 as a stimulant rather than the peptide pool, which may have weakened the cellular response.

In summary, we developed an mRNA vaccine encoding the structural proteins of CHIKV, and showed that it induced a robust immune response in a mouse model. Furthermore, the immunogenicity of homologous booster vaccination was superior to that of Ad5 vaccine and the heterologous immunization regimen evaluated. Thus, the mRNA vaccine against CHIKV constructed in this study has promising potential for development as a CHIKV vaccine candidate.

## Data availability statement

The raw data supporting the conclusions of this article will be made available by the authors, without undue reservation.

## Ethics statement

The animal study was reviewed and approved by The Animal Care and Use Committee in National Institutes for Food and Drug Control.

## Author contributions

YL and BY conceived and supervised the project. JJL and XSL designed the experiments. JJL, XSL, XXL, EF, WL, XHL, and ZZ performed the experiments. JJL, XXL, and JL analyzed the data. WH provided material and technical supports. JJL drafted the first version of the manuscript. HS and YL revised the manuscript. All authors approved the submitted version.

## Supplementary material

Supplementary Table 1 Characterization of mRNA formulation between wild-type (WT) and codon-optimized (OP) vaccines. Quality control parameters were examined after mRNA production.

Supplementary Figure 1 Representative data examined for the wild-type (WT) and codon-optimized (OP) vaccines. Intensity size graphs for the **(A)** WT and **(B)** OP vaccines measured using the dynamic light-scattering method. mRNA content for **(C)** WT and **(D)** OP vaccines tested by Chromatographic graphs.
