# Supplementary figures and images for: Construction and immunogenicity of an mRNA vaccine against chikungunya virus
Source: Front Immunol. 2023 Mar 15;14:1129118. doi: 10.3389/fimmu.2023.1129118 (PMC10050897; doi:10.3389/fimmu.2023.1129118)

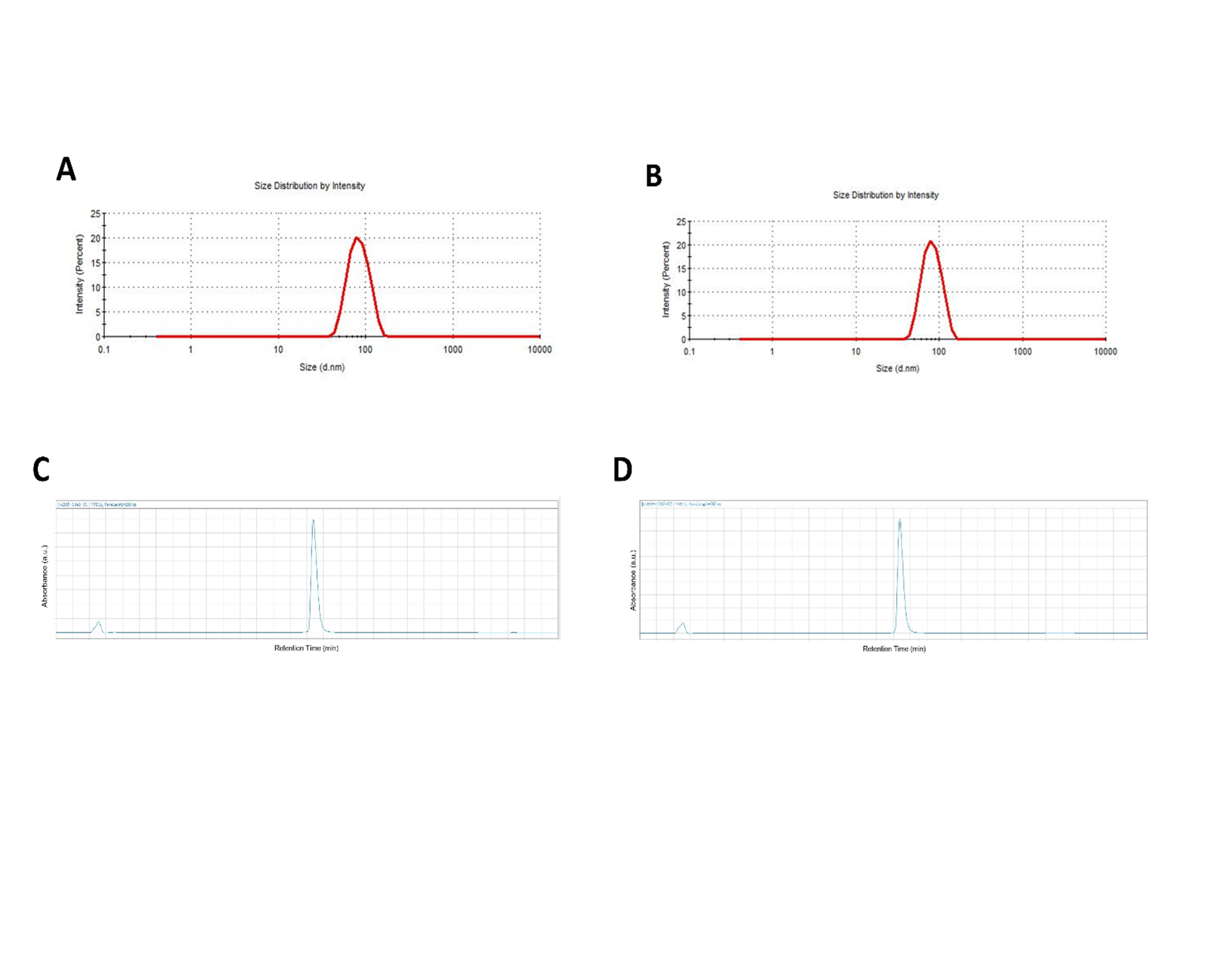

Supplement: Supplementary Figure 1 — Representative data examined for the wild-type (WT) and codon-optimized (OP) vaccines. Intensity size graphs for the (A) WT and (B) OP vaccines measured using the dynamic light-scattering method. mRNA content for (C) WT and (D) OP vaccines tested by Chromatographic graphs. [file Image_1.tif]
